# Child Development Interventions Among Indigenous Peoples in Australia, Canada, New Zealand, and the United States: A Scoping Review

**DOI:** 10.3390/children13020252

**Published:** 2026-02-11

**Authors:** Akilew Awoke Adane, Tracy Reibel, Ailsa Munns, Carrington C. J. Shepherd, Helen D. Bailey, Fiona Stanley, Rhonda Marriott

**Affiliations:** 1 Ngangk Yira Institute for Change, Murdoch University, Murdoch 6150, Australia; akilew.adane@murdoch.edu.au (A.A.A.); tracy.reibel@murdoch.edu.au (T.R.); carrington.shepherd@curtin.edu.au (C.C.J.S.); fiona.stanley@thekids.org.au (F.S.); r.marriott@murdoch.edu.au (R.M.); 2 Curtin Medical School, Faculty of Health Sciences, Curtin University, Bentley 6845, Australia; helen.bailey@curtin.edu.au; 3 Kids Research Institute Australia, The University of Western Australia, Nedlands 6009, Australia

**Keywords:** child development, Indigenous, early intervention, early childhood, cultural safety, social determinants of health, cultural determinants of health

## Abstract

**Background**: Children’s development is dependent on a range of factors influencing their life course outcomes. Protective and challenging social and cultural determinants impact how Indigenous families support their children’s developmental foundations. However, there is a lack of international evidence investigating Indigenous child development interventions. To gain a perspective across nations with comparable settler-colonial histories, this scoping review summarised studies on family and community-centred approaches among Indigenous populations in Australia, Canada, New Zealand, and the United States, focusing on outcomes and evidence gaps. **Methods**: A scoping review followed PRISMA-ScR guidelines. Medline, CINAHL, and PsycINFO (Ovid) were searched from their inception to October 2025, including grey literature sources from Aboriginal HealthInfoNet, the Lowitja Institute and the Secretariat of National Aboriginal and Islander Child Care. Empirical studies, including quantitative, mixed-methods, evaluation studies, and descriptive or case-study designs, were included provided they reported empirical data on intervention outcomes. Due to study heterogeneity, data were synthesised narratively. **Results**: Following screening of 2355 records, eight from 2013 to 2020 met the inclusion criteria. These were mostly small-scale, non-randomising designs evaluating different interventions, with the behavioural and emotional domain being the most frequently assessed outcome, alongside developmental vulnerability and academic/educational areas. There was limited consideration of protective cultural determinants of health in the study design and implementation. Six studies reported positive associations between interventions or programmes and early childhood development outcomes. **Conclusions**: While the number and rigour of identified interventions were limited, several demonstrated potential benefits for Indigenous children’s early childhood development. However, strengthening the evidence base requires culturally grounded, adequately powered evaluations using rigorous study designs that include culturally co-designed adaptations conducted with Indigenous families and communities. Support is recommended for capacity building and funding.

## 1. Introduction

A child’s development is dependent on a range of physical and psychosocial factors, with perinatal and early years’ biology, family and community environments influencing their lifelong developmental health and wellbeing [1,2]. Both protective and challenging social and cultural determinants of health can impact how families are able to support their children’s development. Notably, children’s relationships with significant people in their lives, such as parents, caregivers, extended family and teachers, shape brain development. Adverse stressful circumstances within families and communities have lifelong consequences for learning, socialisation, emotional regulation, and mental health issues, with further implications for education, employment, and maintaining stable relationships. Children who experience issues such as recurrent abuse, chronic neglect, poverty and unsafe communities are particularly vulnerable to toxic stress. Their needs are best met by holistic interdisciplinary approaches focusing on relational care from their families, home visitors, early years care and mental health services within their unique cultural environments. It is recognised that, despite stress resolution, there may be ongoing problems in self-regulation, prosocial behaviours and social-emotional competence [3]. It is also critical to recognise protective factors in the lives of children, their families and communities, including cultural strengths. These relate to the physical and psychosocial attributes and conditions protecting people from the harmful effects of risk factors, thereby enhancing their ability to positively address challenging environments. Protective factors for children may include healthy families, communities and peer relationships, cultural identity, and access to opportunities and health services, particularly mental health assistance [4].

These risk and protective factors are of crucial importance for Indigenous peoples, both nationally and globally, who continue to face significant barriers to health, social equity and self-determination, with their children experiencing disproportionately greater developmental challenges [5]. This is evidenced in the current Australian Early Development Census (AEDC) findings, where approximately 66% of Aboriginal children were shown to have developmental vulnerabilities across five domains of physical health and wellbeing, social competence, emotional maturity, language and cognitive skills (school-based), and communications skills and general knowledge at school entry [6]. Internationally, persistent poorer maternal and developmental health outcomes between the Indigenous and non-Indigenous mothers and infants across developed nations such as Australia, Canada, New Zealand and the United States have been highlighted [7,8,9]. These inequities are shaped by the ongoing legacies of colonialism and racism, which continue to influence key social determinants of health (SDH) through structural discrimination and marginalisation, resulting in entrenched health inequities. SDH are the conditions in which people are born and live, along with their access to power, money, and resources, with primary health care approaches being optimally positioned to address resulting health inequities [10]. Given that lifelong health trajectories are strongly shaped by early maternal and child health and development, recognising and addressing these systematic biases through evidence-based strategies is critical [3,11]. This underscores the urgent need for robust early years interventions and policies that support Indigenous children and families internationally [12].

Investigating pathways to overcome the challenges to designing and implementing effective child development interventions requires a holistic outlook incorporating Indigenous peoples’ worldview of Social and Emotional Wellbeing (SEWB), where social, cultural, political and historical determinants of health strengthen or negatively influence the SEWB domains of family, community, culture, country, and ancestors along with body, mind and spirit [13,14]. In contrast to the predominant deficit-based narrative, which focuses on perpetual problems to be overcome, is the emerging strengths-based approach encompassing cultural determinants of health (CDH). These determinants are foundational to Indigenous cultural identity, functioning as protective factors of health and wellbeing, requiring culturally safe approaches to supporting Indigenous children’s developmental outcomes [15,16]. These need to be developed in partnership with Indigenous and non-Indigenous families and agencies [17].

The challenging and enabling impacts of SDH and CDH, in addition to the use of primary health care, strongly influence individual, family and community inequities and their capacity to respond, and need to be incorporated into the planning and implementation of culturally safe Indigenous child development interventions. As such, working with Indigenous communities encompasses both the social and cultural determinants of health, and when incorporated in comprehensive primary health care, availability will facilitate sustainable and equitable interventions. The principles of primary health care guide structural and environmental programme development and implementation, which include accessible interventions working to alleviate barriers to equal access, use of appropriate technology, cultural safety, intersectoral collaboration, community participation and program sustainability [1].

A significant influence on Indigenous families’ meaningful engagement with children’s mainstream health services is their feelings of anxiety experienced when there is a lack of culturally safe models of care [18,19]. Cultural safety centres on how services and interventions are made available, requiring non-Aboriginal professionals in areas such as health, social services and education to consider their conscious and unconscious biases within their practice and policy decisions in relation to racism [20].

Likely due to the lack of published study outcomes, there is inconclusive evidence on the impacts of risk and CDH protective factors on child development and culturally safe interventions for Indigenous families and communities [16,21]. A notable issue is the difficulties involved with measuring place-based strategies, particularly if there is a lack of researchers with cultural knowledge and/or expertise to implement interventions and collect relevant strengths-based data. This confirms the need for interventions to be developed in partnership with Indigenous peoples to account for varying cultural norms and expectations with flexibility to incorporate each community’s culture and social mores, so these are clearly evident to policymakers, services and practitioners [1]. These approaches include ensuring the availability of culturally safe service delivery using co-designed and culturally adapted interventions which emphasise the CDH. Understanding the drivers of vulnerabilities and protective factors is crucially important to establishing optimal, culturally relevant early interventions for Indigenous child development, which account for the context in which these are being applied [13].

Taking account of all the above evidence, the primary aim of this review is to map and synthesise evidence of child development interventions and their outcomes among Indigenous populations in Australia, Canada, New Zealand and the United States. A secondary analytic lens will critique the cultural validity of the existing evidence base. Understanding the extent of evidence from existing programmes will enable identification of gaps in the knowledge, which in turn will inform future investigations into culturally safe interventions for Indigenous children.

## 2. Methods

As this was a scoping review, the review protocol was not registered. However, the review was conducted in accordance with the PRISMA Extension for Scoping Reviews (PRISMA-ScR) guidelines [22].

### 2.1. Eligibility Criteria

Studies were eligible for inclusion if they met the following criteria. The population of interest was Indigenous children, defined as individuals from birth through 12 years, residing in Australia, Canada, New Zealand or the United States. These developed countries have comparable settler-colonial histories dominated by distinct Western-centric political, territorial, and epistemological phenomena within their health and social care systems [23] (Carey and Silverstein 2020). Within these environments, Indigenous peoples experience persistent social and health challenges, and it is of particular importance to families with children that their early years needs are effectively addressed [3,11,12].

Interventions delivered directly to children, or indirectly through caregivers, families, or communities, for the benefit of child development, were eligible. Eligible interventions included programmes, policies, services, or strategies aimed at improving any domain of child development, including cognitive, socio-emotional, language, motor, behavioural, or broader developmental functioning. We included empirical studies, including quantitative, mixed-methods, evaluation studies, and descriptive or case-study designs, provided they reported empirical data on intervention outcomes, with mechanisms or acceptability extracted if reported but not required. Studies must include a clear comparison group, non-intervention group, or pre-post design to allow assessment of intervention effectiveness. Both peer-reviewed journal articles and grey literature, such as reports, evaluations, or theses, were eligible for inclusion. No time restrictions were applied to the search. However, studies were excluded if they were purely qualitative without any quantitative or evaluative component assessing intervention outcomes. Studies that did not focus on Indigenous populations, or where Indigenous participants could not be clearly distinguished in the results, were also excluded. Research unrelated to child development outcomes, such as studies focusing solely on physical health, nutrition, or service access without developmental measures, was not eligible. In addition, reviews, editorials, commentaries, conference abstracts without full data, protocols, and non-English publications were excluded. While systematic and narrative reviews were not included as primary sources of evidence, their reference lists were screened to identify additional eligible primary studies.

### 2.2. Information Sources

To identify potentially relevant records, Medline, CINAHL, and PsycINFO (Ovid) were searched from their inception to October 2025. In addition to the main databases, grey literature sources included Aboriginal Health Infonet, the Lowitja Institute, and SNAICC (the Secretariat of National Aboriginal and Islander Child Care), to ensure comprehensive coverage of relevant documents.

### 2.3. Search

The search strategy combined terms related to Indigenous populations, child development interventions, and developmental outcomes, using a wide range of keywords for each concept. The full electronic search strategies for each database are available in the Supplemental File (Table S1).

### 2.4. Selection of Sources of Evidence

All records identified were imported into an EndNote library, and duplicate records were removed. One author (AAA) first screened the titles to exclude clearly irrelevant records. Records that passed the title screening were then independently assessed by two authors (AAA and AM/TR) based on the abstracts. Full-text evaluation was subsequently conducted independently by the same authors using the predefined eligibility criteria. Any disagreements were resolved through face-to-face discussion.

### 2.5. Data Extraction and Synthesis

We employed a standardised Excel data abstraction template to extract relevant variables and information from all eligible studies. One author (AA) charted data from every included study, while TR and AM independently verified and double-checked the entries against the original studies to ensure accuracy.

The standardised template included key study characteristics, sample details, intervention features, outcomes, and other relevant information. Study-level data included authors and year, study period, country, Indigenous group, setting, design, aims, and inclusion and exclusion criteria. Sample characteristics comprised participants’ age, sample size, and relevant characteristics. Intervention details included intervention name, type, description, duration, frequency, comparator, and delivery personnel. Child outcomes were recorded by outcome type, measures used, and a summary of findings and effect size. Additional items included cultural adaptation and Indigenous involvement.

Given the purpose and scope of this review, we undertook a qualitative synthesis of the included studies. Tables were used to summarise key study characteristics, interventions, child outcomes and principal findings. Due to the small number of eligible studies, the findings were narratively synthesised, and it was not feasible to group or summarise them by child outcome domain, intervention type, study design, or other key characteristics.

## 3. Results

### 3.1. Study Identification and Selection

**Figure 1:**
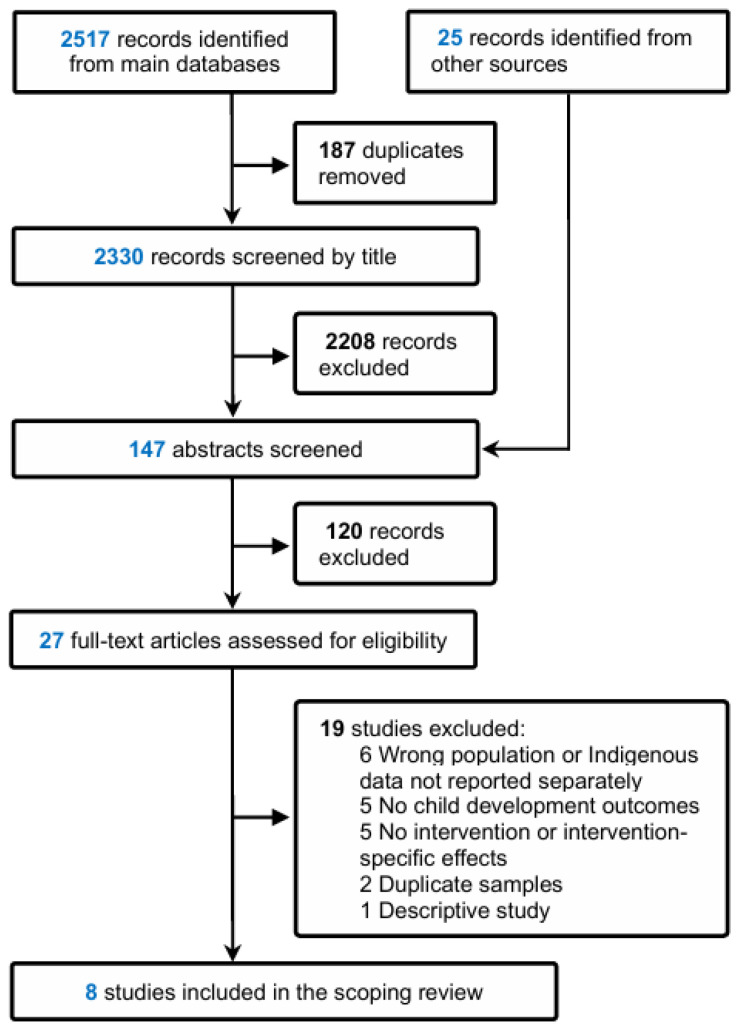
PRISMA flowchart for study selection.

We identified 2517 records through database searches and removed 187 duplicates, leaving 2330 titles for screening. An additional 25 records were located through other sources. In total, 147 abstracts and 27 full-text articles were independently reviewed. Of the 27 studies evaluated by full-text, 19 were excluded for various reasons, such as focusing on non-Indigenous populations or not reporting Indigenous data separately, lacking child development outcomes, or not reporting an intervention or intervention-specific effects. Full-text articles that were assessed but excluded, along with the reasons for exclusion, are provided in Table S2. The remaining eight studies [24,25,26,27,28,29,30,31] met the eligibility criteria and were included in the scoping review (Figure 1).

### 3.2. Characteristics of Included Studies

Key characteristics of eligible studies, including study country, Indigenous group, study design, child age, and sample size, are summarised in Table 1. Half of the studies were conducted in Australia (two in the Northern Territory, one in Western Australia, and one national), with the remainder from the United States (one in Arizona, one in the North-West region as defined by the Indian Health Service and one in Oklahoma) and Canada (one in Manitoba); none were found in New Zealand. Study designs included randomised controlled trials (RCTs), quasi-experimental studies, cohort (based on linked administrative data) studies and descriptive pre–post evaluations, targeting children from infancy through to early school age. Sample sizes ranged from small pre-post evaluations with around 30 participants to large population-based cohorts of more than 8000 children (Table 1).

### 3.3. Intervention Effects on Child Development

The interventions included culturally adapted parenting programmes (Indigenous Triple P, Family Spirit, Promoting First Relationships), early childhood education initiatives (Universal Pre-K, Mobile Preschool Programme, Exploring Together Preschool Program), a prenatal cash transfer program (Healthy Baby Prenatal Benefit), and community playgroup participation. Further details of interventions or programmes are available in Table S3. Child outcomes assessed were social–emotional/behavioural, global development or school readiness, academic/cognitive, and physical (motor) development (Table S4). Indigenous involvement varied, with some interventions fully co-designed and culturally adapted, and others showing limited or no Indigenous engagement. The effects of these eight interventions or programmes, as reported in the included studies, are qualitatively summarised below and in Table 2.

As shown in Table S4, all studies assessed social-emotional or behavioural outcomes except the one by Gormley et al. [28], which evaluated academic outcomes or cognitive skills. A few studies also evaluated other domains, including global development, school readiness, and physical (motor) development. These studies used a range of standardised measures, including the Eyberg Child Behaviour Inventory (ECBI), Child Adjustment and Parent Efficacy Scale—Developmental Disability (CAPES-DD), Infant–Toddler Social and Emotional Assessment (ITSEA), Strengths and Difficulties Questionnaire (SDQ), Woodcock–Johnson Tests, the Ngari-P (a culturally specific tool developed on the Tiwi Islands) [32] and the Early Development Instrument (EDI) or its Australian adaptation, the Australian Early Development Census (AEDC).

Among the four culturally adapted interventions [24,25,26,30], three showed significant improvements in child behavioural and emotional outcomes. While the study by Booth-LaForce et al. [26] may have been underpowered to demonstrate statistical significance, this study showed improvements in emotional or behavioural outcomes, specifically in internalising, externalising and competence domains of the ITSEA. Additionally, Robinson et al. [30] observed reductions in problem behaviours among remote Tiwi Indigenous children but not for the urban Indigenous subgroup, likely due to a smaller sample size. All interventions demonstrated elements identified for authentic cultural adaptation through strong emphasis on working in partnership with Indigenous communities, inclusion of CDH and facilitating Indigenous-led programme implementation [33], which also highlight many aspects of primary co-design strategies [34,35].

Among interventions without cultural adaptation or where adaptation was not required, the largest linked data study (*n* = 8209), and only Canadian study [27] evaluated the Healthy Baby Prenatal Benefit (an unconditional prenatal income supplement). This programme reduced developmental vulnerability in Language and Cognitive Skills, Communication Skills and General Knowledge but showed no significant effects on Social Competence, Emotional Maturity, or Physical Health and Wellbeing domain of the EDI. Nutton [29] (Mobile Preschool Programme in the Northern Territory, Australia) reported reduced overall developmental vulnerability for children with full-year or high attendance, and improvements in Social Competence, Communication Skills, General Knowledge, and Physical Health and Wellbeing. As part of the Longitudinal Study of Indigenous Children study, Williams and colleagues [31] evaluated the effect of playgroup participation on children’s functional motor skills, social–emotional development and expressive vocabulary. They found no direct effects on these outcomes, though an indirect association with the expressive vocabulary was observed via parent home-learning. Finally, in a quasi-experimental study evaluating a universal pre-kindergarten program in the state of Oklahoma, USA, Gormley et al. [28] reported improvements in letter–word identification and spelling, whereas applied problem scores and comparisons between half- and full-day programmes were mostly not statistically significant.

## 4. Discussion

This scoping review aimed to synthesise evidence on child development interventions for Indigenous populations in Australia, Canada, New Zealand and the United States, and identify key evidence gaps. Although Indigenous children globally are more likely to experience developmental challenges, largely due to their social and cultural determinants not being fully supported, and despite the expectation that many programmes exist to support them, we identified surprisingly few studies that evaluated interventions targeting early childhood development in the four countries, with none identified from New Zealand.

Overall, while the number of identified programmes and interventions was very limited, several demonstrated potential benefits for Indigenous children’s early childhood development. However, the strength of the evidence is constrained by small-scale sizes and the predominance of non-RCT or quasi-experimental study designs, which are vulnerable to selection bias, unmeasured confounding and limited generalisability. Strengthening the evidence base will require culturally grounded, adequately powered evaluations using more rigorous, Indigenous-informed study designs. The ability to develop and implement culturally safe intersectoral approaches to Indigenous programmes rests on cultural co-design and/or adaptation, which enable Indigenous voices, values and worldviews to determine processes for design, implementation and evaluation of strategies. Equitable and shared decision making through co-design also addresses power differentials often present in agencies such as health, social services and education. Through the insight of lived experiences, local cultural knowledge and expertise about the needs of their own geographical and social areas, Indigenous individuals and families are best placed to identify and direct activities of value to themselves and their communities. When conducted authentically, these approaches facilitate equitable partnerships between Indigenous and non-Indigenous teams, thereby supporting their empowerment and self-determination [33,35,36]. An Australian study has developed six key evidence-based principles for successful co-design with Aboriginal and Torres Strait Islander people and communities.

1. Focus on First Nations leadership
2. Maintain a culturally grounded approach
3. Promote respect
4. Promote inclusivity
5. Ensure community benefit
6. Ensure transparency and rigorous evaluation [34,35].

In contrast, cultural adaptation relates to reviewing and changing the structure of an evidence-based programme to ensure the cultural requirements of a discrete cultural community are met, with the emphasis on partnership approaches, worldviews and CDH [33].

Further, in relation to process and impact evaluation of programmes, caution is needed to ensure the design is Indigenous-informed and led, measures elements or factors which are meaningful to Indigenous peoples and are not tokenistic. This also requires Indigenous evaluators and Indigenous lenses used in data analysis, all critical components of a culturally safe approach [34,35,37]. Of note is the need to ensure outcome measures have been validated for use with Indigenous populations. Study evaluations that conform to a dominant Western societal expectation and do not present authentic, relevant results for Indigenous people and their communities do little to support Indigenous self-determination. It has been established that assessment of Indigenous children’s development can be compromised due to measurement of progress being frequently undertaken using universal Western-centric norms in addition to a lack of knowledge of their diverse lived experiences across urban, rural and remote communities [1,38].

There is also increasing awareness of the need for non-Indigenous researchers to review their concepts of research rigour and adjust their quality appraisal of data to be responsive to Indigenous peoples’ worldviews. Ensuring cultural rigour requires consideration of how engagement is undertaken with Indigenous peoples and communities to confirm the cultural validity of research results. Additionally, decisions in relation to what data is important and relevant to collect, along with methods of collection, are crucial aspects of co-design contributing to the rigour of Indigenous-led studies [35].

A secondary analytic lens critiqued the cultural validity of the research. Evidence has clearly established the importance and significance of culturally safe interventions and engagement, with Indigenous SEWB and CDH being central to the effectiveness of strategies [13,14]. However, of the eight studies in the review, only four (50%) [24,25,26,31] were culturally adapted and co-designed with Indigenous peoples, with one (12.5%) [30] having partial adaptation and Indigenous involvement with design. Of these interventions, two were conducted in the USA [25,26] and three in Australia [24,30,31]. These strategies support the need for development of culturally safe interventions integrating CDH through partnership approaches. This is a critical aspect in enhancing Indigenous child development through authentic recognition of risk and protective factors, including CDH, within SEWB domains [13,14,16,17,21]. Importantly, this scoping review identified that half of the identified interventions did not incorporate these culturally safe approaches.

A range of outcomes was identified in each study, with the Behavioural and Emotional domain most frequently measured (4/8, 50%), both in addition to developmental vulnerability in relation to language, cognition, physical health, social competence, emotional maturity, communication and general knowledge (3/8, 37.5%) and academic/educational (1/8, 12.5%). The predominance of behavioural and emotional intervention programmes highlights the importance being placed on children’s abilities to cope with stress and toxic stress caused by a range of home, societal and environmental impacts [3]. Importantly, all identified studies in this domain were supported by comprehensive or partial cultural adaptation and co-design, ensuring Indigenous peoples have their CDH, values and worldviews understood and incorporated into meaningful and acceptable program design, implementation and evaluation outcomes [35].

Findings from three of these four programmes were positively associated with reductions in problem behaviours with more effective self-regulation, which is strongly linked to culturally safe intervention approaches [3]. Strategies designed to address developmental vulnerabilities included social competence and emotional maturity measured in behavioural and emotional programmes, exploring how these impacted the development of cognition, language/communication, general knowledge and physical health and wellbeing. Cultural safety was not obviously present in two of the interventions, as no cultural adaptation of the intervention was undertaken. However, one had partial Indigenous involvement, evidenced by part-time employment and training of local Indigenous coordinators and teaching assistants in a very remote Australian preschool program [29]. Outcomes in these studies also demonstrated a decreased likelihood of vulnerability in language/communication and general knowledge, with mixed outcomes for physical health and wellbeing, social competence and emotional maturity. The lack of consistent findings across these latter domains may reflect differences in study populations and the nature of the interventions, despite both studies using similar child development measures. Additionally, without the inclusion of CDH, known as protective factors within these interventions, it can be suggested that minimal Indigenous participation influenced the lack of consensus on the outcomes [3,4,17].

Findings from the sole academic/educational intervention suggested enhanced outcomes for word identification, spelling and applied problems, but the study lacked statistically significant power [28]. Similarly, the only programme addressing functional motor skills in addition to social-emotional competence and expressive vocabulary had no direct associations, but a suggestion of indirect improvements to expressive vocabulary [31]. These mixed findings are likely driven by differences in study populations, program focus, delivery setting and intensity. The programmes also varied substantially in the child outcomes assessed and the measures used, which likely contributed to the observed pattern of results.

All studies used universally accepted, validated measures. However, it is not specified if these were based on specific Indigenous cultural child developmental norms. This is of particular importance for culturally relevant, meaningful assessments and subsequent evidence-based programme improvements. Interventions supporting children’s development need to reflect diversity and decolonising approaches, enhancing Indigenous knowledge and skills [1,38]. Through partnership approaches with Indigenous consumers, practitioners and agencies, only four of the eight study interventions had comprehensive cultural adaptations [1]. Interdisciplinary practitioners and agencies must plan and implement authentically Indigenous-informed, culturally safe interventions encompassing SEWB domains, CDH and self-determination.

A crucial consideration for child development interventions is the ability to assess and address the SDH impacting families and communities where these activities are taking place. Seven included studies documented SDH factors influencing participants’ social environments, with one [28] having only minimal data in this domain. Understanding social contexts reduces the risk of inappropriate programme delivery. Of note was the range of interventions undertaken in rural and remote areas. Four were delivered in rural and remote areas, with two undertaken across urban, rural and remote locations. When engaging with rural and remote populations, practitioners need to consider the long-term sustainability of their interventions, taking into account factors such as staffing, travelling distance and high costs, particularly when government and non-government supporting or start-up funding has ceased [1]. It is strongly recommended that the principles of comprehensive primary health care are incorporated into program and financial planning to help address these issues of equity, affordability and longevity [1].

Overall, there were difficulties in evaluating the systemic reasons contributing to evidence gaps within the included studies. There has been long term disengagement from Western-centric research by Indigenous groups, where investigators have not developed culturally safe approaches [34,35,37]. Funding issues have also negatively contributed to the development, implementation and evaluation of Indigenous research [1]. However, these were not clarified within the studies.

## 5. Limitations

This study has several notable limitations. Despite a comprehensive search, very few studies were found, most of which were small-scale and evaluated different programmes or interventions. Importantly, there was a research gap with no studies meeting the search criteria identified in the last five years (2021–2025). Additionally, non-randomised study designs predominated, limiting causal inference. Notably, there was limited Indigenous involvement in study design or implementation. There is an epistemological tension between advocating for Indigenous knowledge systems and privileging quantitative outcome measures. This does not suggest that the focus on quantitative evidence in this scoping review is the only credible form of knowledge, particularly in Indigenous contexts. Overall, our search strategy may not have captured all relevant studies, with only English-language studies from four countries included, and no eligible studies identified from New Zealand, potentially due to the absence of manual searches of Māori-specific repositories. Due to the lack of study collaborators from Canada, New Zealand and the USA, it is possible that relevant grey literature was not identified. However, it is unlikely that key studies were missed. Moreover, due to the limited number of studies and substantial heterogeneity in interventions and outcome measures, it was not possible to systematically analyse the relationship between cultural adaptation and intervention outcomes.

## 6. Implications and Future Directions

This scoping review identified an urgent need for co-designed, locally relevant interventions and programmes to address inequities present in Indigenous children’s developmental outcomes in the four countries of interest. We acknowledge that existing programmes are highly likely to be under-reported, which indicates the requirement for better documentation and/or publication of funded initiatives and broad dissemination of findings. This, in turn, requires support in the form of adequate funding for Indigenous communities to build their research, implementation and evaluation capacity, as it is probable many effective interventions lack time for scientific evaluation. As such, it is recommended that national health and education departments establish dedicated funding streams to support intervention development and evaluation projects led by Indigenous communities and employing mixed-methods designs to ensure the rigour required to build evidence and the sustainability of effective interventions. As there was an identified need to incorporate cultural safety into interventions, a further recommendation is to incorporate cultural safety training and co-design principles into core competency standards for primary health, public health, social support and early childhood education practitioners.

As we were unable to locate any New Zealand studies, there is an imperative for research focusing on Māori children’s early years development. There is also an urgent need to develop and validate child development assessment tools for use by diverse Indigenous populations, which include SEWB indicators [1,38]. Investigation into the use of the Ngari-P tool is recommended, noting its positive association with child behaviour by Robinson et al. [30]. There are also opportunities to promote research for Indigenous child development interventions through structural policies such as unconditional cash transfers for research participation, which address the issues of potential power imbalances [39].

## 7. Conclusions

This scoping review has synthesised the limited evidence in an effort to identify and critically review evidence-based Indigenous child development interventions. The scoping review identified a limited scope of programmes across Australia, Canada, and the United States, with no reported interventions from New Zealand. Study findings have highlighted the need for culturally safe approaches in the planning, delivery and evaluation of interventions for Indigenous children, supported by co-designed and/or culturally adapted programmes, and funded for realistic periods of time and with appropriate evaluation strategies to measure outcomes. Without these approaches, interventions will continue to be piecemeal and lack the evidence required to scale up or adapt for other similar populations. Central to this is understanding the influence of the SDH and CDH on the design of interventions, with implementation of primary health care approaches needed to facilitate affordable, accessible and sustainable interventions using appropriate technology and intersectoral collaboration to effectively incorporate cultural strengths and address structural and systematic challenges [1,10]. These changes are needed to enable non-Indigenous government and non-government agencies to recognise Indigenous culture as credible knowledge, with CDH included in community program development. Cultural determinants critically inform co-designed models of practice for culturally safe Indigenous child development interventions across a range of urban, rural and remote locations and are needed to address adverse SDH such as racism, marginalisation and health and educational inequities [3,11,15]. Contemporary evidence-based, co-designed approaches are vital for authentic and sustainable early years developmental interventions for Indigenous families, children and their communities.

## Figures and Tables

**Table 1:** Summary of key characteristics of included studies.

Study	Country	Indigenous Group	Study Design	Child Age (Year)	Sample Size
**Andersson 2020** [24]	Australia	Aboriginal	Pre-post	NA	30
**Barlow 2015** [25]	USA	American Indian	RCT	3	322
**Booth-LaForce 2020** [26]	USA	American Indian and Alaska Native	RCT	10–30 months	34
**Enns 2021** [27]	Canada	First Nations	Cohort (linked data)	~5	8209
**Gormley 2005** [28]	USA	Native American	Quasi-experimental	4–5	240
**Nutton 2013** [29]	Australia	Remote Indigenous	Cohort (linked data)	~5	115
**Robinson 2009** [30]	Australia	Tiwi and Urban Indigenous	Quasi-experimental	4–6	126
**Williams 2017** [31]	Australia	Aboriginal and Torres Strait Islander	Cohort (follow-up)	4	622

*NA, not available.*

**Table 2:** Summary of intervention characteristics, child outcomes and key findings.

Study	Intervention	Duration/Delivery	Cultural/Indigenous Involvement	Outcome Domain(s) and Measure(s)	Key Findings
**Andersson 2020** [24]	Indigenous Triple P: Parenting programme with simplified materials, “Parent Packs”	Flexible delivery: community hubs, home visits, group sessions	Culturally adapted; co-designed	Behaviour (ECBI); Emotional/Behavioural (CAPES-DD)	↓ Problem behaviour intensity (*p* = 0.013); ↓ Number of problem behaviours (*p* < 0.001); ↑ Prosocial behaviours (*p* = 0.003)
**Barlow 2015** [25]	Family Spirit Intervention: Home-visiting programme targeting positive parenting	43 lessons from late pregnancy to 36 months postpartum, ≤1 h each, tapering schedule	Culturally adapted; co-designed	Emotional and Behavioural (ITSEA)	↑ Externalising, internalising, and dysregulation; NS change in competence
**Booth-LaForce 2020** [26]	Promoting First Relationships: Caregiver-child relationship home visits	10 visits over ~15 weeks	Culturally adapted; co-designed	Emotional and Behavioural (ITSEA)	No significant differences in any child behaviour domains
**Enns 2021** [27]	Healthy Baby Prenatal Benefit: Unconditional cash transfer ($81/month)	2nd and 3rd trimester	Not adapted; no Indigenous involvement	Developmental vulnerability (EDI)	↓ Likelihood of vulnerability in Language and Cognitive Development, Communication, and General Knowledge No significant change in Physical Health and Well-Being, Social Competence or Emotional Maturity
**Gormley 2005** [28]	Universal Pre-K: Oklahoma state-funded pre-kindergarten	1 academic year, 56% half-day	Not adapted; no Indigenous involvement	Academic/Educational (Woodcock–Johnson Tests)	↑ Letter–Word Identification and Spelling; Applied Problems ↑, but NS; half vs. full day, mostly NS
**Nutton 2013** [29]	Mobile Preschool Programme: Early learning, school-readiness, developmental support	Evaluated by availability, attendance, and quality	No adaptation; partial Indigenous involvement	Developmental vulnerability (AEDC)	Full access ↓ vulnerability (25% vs. 69%); high attendance ↓ vulnerability (26% vs. 56%); high-quality ↑ Physical Health and Well-Being, Social Competence and Communication and General Knowledge
**Robinson 2009** [30]	Exploring Together Preschool: Early intervention, parent–child sessions	10 weekly 2 h group sessions	Partial adaptation; partial Indigenous involvement	Behaviour and Adjustment (Ngari-P, SDQ)	↓ Problem behaviours in both groups, but NS for the urban group
**Williams 2017** [31]	Playgroup attendance	Waves 2 and 3; assessed supported vs. unsupported groups	Culturally adapted; partial Indigenous involvement	Functional motor skills, Social-emotional (SDQ), Expressive vocabulary (Renfrew Word Finding Test)	No direct associations; indirect ↑ expressive vocabulary via parent home-learning

*↑, improvement; ↓, reduction; NS, not statistically significant. AEDC, Australian Early Development Census; CAPES-DD, Child Adjustment and Parent Efficacy Scale* *—* *Developmental Disability; ECBI, Eyberg Child Behaviour Inventory; EDI, Early Development Instrument; ITSEA, Infant* *–* *Toddler Social and Emotional Assessment; SDQ, Strengths and Difficulties Questionnaire.*

## Data Availability

Data is contained within the article or Supplementary Materials.

## Supplementary Materials

The following supporting information can be downloaded at: https://www.mdpi.com/article/10.3390/children13020252/s1, Table S1. Main database search strategies and results. Table S2. Full-text articles excluded and reasons for exclusion. Table S3. Details of interventions or programs across included studies. Table S4. Child outcomes of included studies. References [40,41,42,43,44,45,46,47,48,49,50,51,52,53,54,55,56,57,58] are cited in the supplementary materials.
