# Supplementary material for: Child Development Interventions Among Indigenous Peoples in Australia, Canada, New Zealand, and the United States: A Scoping Review
Source: Children (Basel). 2026 Feb 11;13(2):252. doi: 10.3390/children13020252 (PMC12939623; doi:10.3390/children13020252)
Supplement: Supplementary file 1 [file children-13-00252-s001.zip › children-4114290-supplementary.pdf]

Table S1. Main database search strategies and results

S.No	MEDLINE (Ovid) Search Strategy	Results
	Population – Indigenous People	
1	exp Indigenous Peoples/ OR exp Oceanic Ancestry Group/ OR exp Indians, North American/ OR (Aborigin* OR Indigenous OR Inuit OR Metis OR native OR "First Nation*" OR "First people*" OR "Torres Strait" OR Maori OR Polynesian* OR "Oceanic ancestry group" OR "American Indian" OR "Pacific Island*").mp.	376775
	Intervention – Programmes / Services	
2	exp Parenting/ OR exp Parent-Child Relations/ OR exp Child, Preschool/ OR exp Early Intervention, Educational/ OR exp Health Promotion/ OR exp Health Education/ OR ("parenting program*" OR "parent support" OR "peer support" OR "family support program*" OR "parenting intervention*" OR "early childhood program*" OR "early learning" OR "early childhood education" OR "school readiness program*" OR "community-based program*" OR "culturally adapted intervention*" OR "Indigenous-led program*" OR "home visiting program*" OR "child development program*" OR "developmental intervention*" OR "psychosocial intervention*" OR "parent-child program*" OR "co-design*" OR "co-designed" OR "culturally safe" OR "cultural safety").mp.	1399796
	Outcome – Child Development	
3	exp Child Development/ OR exp Growth/ OR exp Cognition/ OR exp Language Development/ OR exp Psychomotor Performance/ OR exp Academic Performance/ OR exp Child Health/ OR ("child development" OR "child growth" OR "cognitive development" OR "language development" OR "socioemotional development" OR "motor development" OR psychomotor OR "academic performance" OR "child wellbeing" OR "child health" OR "social and emotional wellbeing").mp.	1451248
4	1 AND 2 AND 3	1767
5	Limit #4 to English Language	1715
	CINAHL Ultimate Search	
	Population – Indigenous Peoples	
1	(MH "Indigenous Peoples" OR MH "Oceanic Ancestry Group" OR MH "American Indian" OR Aborigin* OR Indigenous OR Inuit OR Metis OR native OR "First Nation*" OR "First people*" OR "Torres Strait" OR Maori OR Polynesian* OR "Pacific Island*")	62,299
	Intervention – Programmes / Services	
2	(MH "Parenting" OR MH "Parent-Child Relations" OR MH "Child, Preschool" OR MH "Health Promotion" OR MH "Health Education" OR MH "Early Intervention, Educational" OR "parenting program*" OR "parent support" OR "peer support" OR "family support program*" OR "parenting intervention*" OR "early childhood program*" OR "early learning" OR "early childhood education" OR "school readiness program*" OR "community-based program*" OR "culturally adapted intervention*" OR "Indigenous-led program*" OR "home visiting program*" OR "child development program*" OR "developmental intervention*" OR "psychosocial intervention*" OR "parent-child program*" OR "co-design*" OR "co-designed" OR "culturally safe" OR "cultural safety")	414,323
	Outcome – Child Development	
3	(MH "Child Development" OR MH "Cognition" OR MH "Language Development" OR MH "Psychomotor Performance" OR MH "Academic Performance" OR MH "Child Health" OR "child development" OR "child growth" OR "cognitive development" OR "language development" OR "socioemotional development" OR "motor development" OR psychomotor OR "academic performance" OR "child wellbeing" OR "child health" OR "social and emotional wellbeing")	205,154
4	1 AND 2 AND 3	742

[OFFICIAL]

5	Limit #4 to English Language	738
	PsycINFO (Ovid) Search	
	Population – Indigenous Peoples	
1	tiab(Aborigin* OR Indigenous OR Inuit OR Metis OR native OR "First Nation*" OR "First people*" OR "Torres Strait" OR Maori OR Polynesian* OR "Pacific Island*")	54,457
	Intervention – Programmes / Services	
2	tiab("parenting program*" OR "parent support" OR "peer support" OR "family support program*" OR "parenting intervention*" OR "early childhood program*" OR "early learning" OR "early childhood education" OR "school readiness program*" OR "community-based program*" OR "culturally adapted intervention*" OR "Indigenous-led program*" OR "home visiting program*" OR "child development program*" OR "developmental intervention*" OR "psychosocial intervention*" OR "parent-child program*" OR "co-design*" OR "co-designed" OR "culturally safe" OR "cultural safety")	30,514
	Outcome – Child Development	73,932
3	tiab("child development" OR "child growth" OR "cognitive development" OR "language development" OR "socioemotional development" OR "motor development" OR psychomotor OR "academic performance" OR "child wellbeing" OR "child health" OR "social and emotional wellbeing")	
4	1 AND 2 AND 3	64

Table S2. Full-text articles excluded and reasons for exclusion.

**Study**	**Reason/s for exclusion**
SNAICC 2025 [40]	No child development outcomes
SNAICC 2025 [41]	No child development outcomes
Aflague 2021 [42]	Indigenous data not reported separately
Bromley 2022 [43]	No intervention or intervention-specific effect reported.
Bruner 2025 [44]	Wrong population
Femald 2017 [45]	Wrong population
Haroz 2019 [46]	No intervention or intervention-specific effect reported.
He 2021 [47]	No intervention or intervention-specific effect reported.
Karanth 2010 [48]	Wrong population
Khan 2014 [49]	Descriptive
Liddell 2011 [50]	Wrong population
Neha 2020 [51]	No intervention or intervention-specific effect reported.
Parletta 2013 [52]	Wrong population
Raman 2017 [53]	No intervention or intervention-specific effect reported.
Robinson 2012 [54]	Duplicate sample as another included study
Robinson 2013 [55]	Duplicate sample as another included study
SNAICC 2023 [56]	No child development outcomes
Walkup 2009 [57]	No child development outcomes
Williamson 2023 [58]	No child development outcomes

Table S3. Details of interventions or programs across included studies.

Study	Intervention / Program Name	Intervention type	Description	Duration	Frequency / Intensity	Comparator (control group)	Delivery personnel
Andersson 2020 [24]	Indigenous Triple P (Level 4 + Stepping Stones) known as Jandu Yani U project	Parenting/family-support program	Culturally adapted Triple P for parents (including children with developmental disabilities), using low-literacy materials and "Parent Packs." Delivered flexibly via hubs or home visits with ongoing mentoring. Implemented 2017–2019 with data at pre-, post-, and follow-up.	Not specified	Group sessions; brief advice (Level 1–2) also delivered to community	None (pre-post design)	Local Aboriginal parent coaches trained in Triple P, supported by non-Indigenous trainer and Aboriginal consultant; ongoing peer mentoring
Barlow 2015 [25]	Family Spirit Intervention	Home-visiting, culturally tailored parenting and maternal health program	A structured, culturally congruent, home-based program of 43 one-on-one lessons focusing on positive parenting, reducing harsh/coercive practices, supporting maternal wellbeing, and addressing risk behaviours. Uses illustrated flip-charts and a standard session format.	From pregnancy (third trimester) to 36 months postpartum	Weekly (3rd trimester); Biweekly (0–4 months); Monthly (4–12 months); Bimonthly (12–36 months) Sessions ≤1 hour	Optimised standard care (transport to visits, information pamphlets, referrals; delivered by non-intervention staff)	Family health educators (Native paraprofessionals) with: High school diploma or GED ≥2 years job-related education/experience Fluency in Native language and English
Booth-LaForce 2020 [26]	Promoting First Relationships (PFR) program	Strengths-based, home-visiting preventive intervention focusing on caregiver-child relationship	10 home-visits delivered to Immediate group; culturally adapted based on community focus groups and staff input	the median time between baseline and follow-up assessments was 15 weeks	10 visits	Wait-list control group	Home-visiting staff affiliated with the project and tribal/community partners
Enns 2021 [27]	Healthy Baby Prenatal Benefit (HBPB)	Cash transfer / unconditional prenatal income supplement	Up to CAD $81/month for low-income pregnant women to support prenatal health and early child development	2nd and 3rd trimester of pregnancy	Monthly cash payments	Eligible women who did not receive HBPB	Administrative delivery via provincial income assistance system (not personnel-based)
Gormley 2005 [28]	Universal Pre-K (Tulsa Public Schools)	Universal state-funded pre-	Oklahoma state-funded pre-K program for 4-year-olds, delivered by certified early	One year of pre-K (the academic year)	Full-year school pre-K (attendance	Children just starting Tulsa pre-K at	Public school teachers in Tulsa

[OFFICIAL]

		kindergarten (pre-K)	childhood teachers with bachelor’s degrees; provides pre-K education before kindergarten entry		during school year), with ~56% of the state’s pre-K programs were half day	testing (vs those who completed it the previous year).	
Nutton 2013 [28]	Mobile Preschool Program	Mobile, outreach early childhood education service	Preschool delivered via mobile units to very remote Aboriginal communities, providing early learning, school-readiness, pre-literacy/numeracy activities, developmental stimulation, and transition support. Evaluation compared groups by availability (limited ≤191 days; full ≥192 days), attendance (low ≤79 days; high ≥80 days), and program quality	Program operated across 18 sites. Core-funded sites: operating ≥4 years. Closing-the-Gap sites: operating 10–18 months. Some sites had intermittent closures due to teacher recruitment challenges.	Varied by community and visit schedule; key exposure measure = number of days attended.	Varied by community and visit schedule; primary exposure = number of days attended	Mobile preschool teacher – travelled to communities, provided supervision, modelling, curriculum support; visited each site about one day per fortnight. Assistant teachers (local Aboriginal staff) – delivered day-to-day preschool activities with ongoing mentoring and skills development.
Robinson 2009 [30]	Exploring Together Preschool Program (ETPP)” under the Let’s Start initiative	Early-intervention / parent-child + preschool program for preschool-aged children and families	Group-based early-intervention program for preschool children and their parents, focusing on parent–child interaction, social skills, emotional regulation, behaviour management, and school-readiness. Culturally adapted for Indigenous communities.	10 weeks	Weekly 2-hour sessions (children’s group + parent group + joint parent–child sessions)	pre–post comparisons	Trained facilitators including Indigenous and non-Indigenous staff; early childhood specialists; school and community workers
Williams 2017 [31]	Playgroup attendance	Playgroup participation	Playgroup participation based on parent report at Waves 2 and 3 (yes/no). Parents also indicated whether the group had a facilitator (yes/no), identifying supported playgroups	Not specified	Not specified	No playgroup attendance	Parents and paid facilitator

[OFFICIAL]

Table S4. Child outcomes of included studies.

Study	Behavioural/ Social–Emotional	Development / School Readiness	Cognitive / Academic	Physical (motor) development
Andersson 2020 [24]	✓			
Barlow 2015 [25]	✓ *			
Booth-LaForce 2020 [26]	✓			
Enns 2021 [27]	✓	✓	✓	✓
Gormley 2005 [28]			✓	
Nutton 2013 [28]	✓	✓	✓	✓
Robinson 2009 [30]	✓			
Williams 2017 [31]	✓		✓	✓

**(includes emotional & dysregulation*
